# Elaiophylin Inhibits Tumorigenesis of Human Lung Adenocarcinoma by Inhibiting Mitophagy via Suppression of SIRT1/Nrf2 Signaling

**DOI:** 10.3390/cancers14235812

**Published:** 2022-11-25

**Authors:** Jiali Ji, Ke Wang, Xinmin Meng, Hongqin Zhong, Xiyue Li, Hongqing Zhao, Guijuan Xie, Yunying Xie, Xun Wang, Xue Zhu

**Affiliations:** 1 Department of Respiratory and Critical Care Medicine, The Affiliated Wuxi No. 2 People’s Hospital of Nanjing Medical University, Wuxi 214002, China; 2 National Health Commission (NHC) Key Laboratory of Nuclear Medicine, Jiangsu Key Laboratory of Molecular Nuclear Medicine, Jiangsu Institute of Nuclear Medicine, Wuxi 214063, China; 3 Department of Radiopharmaceuticals, School of Pharmacy, Nanjing Medical University, Nanjing 210000, China; 4 Department of Clinical Laboratory, Cancer Hospital of Guangxi Medical University, Nanning 530021, China; 5 Department of Respiratory and Critical Care Medicine, Wuxi Clinical College Affiliated to Nantong University, Wuxi 214002, China; 6 Institute of Medicinal Biotechnology, Chinese Academy of Medical Sciences and Peking Union Medical College, Beijing 100050, China

**Keywords:** lung adenocarcinoma, elaiophylin, mitophagy, oxidative stress, SIRT1/Nrf2 signaling

## Abstract

**Simple Summary:**

Lung adenocarcinoma (LADC) is the pathological type with the highest morbidity and mortality among lung cancers. Although achievements in new therapeutic approaches have been developed, chemotherapy is still the most widely choice for control of LADC. However, the increasing drug resistance becomes the major challenge, so the development of the novel and efficient chemotherapeutic drug is still urgent. Elaiophylin, a new type of autophagy inhibitor, has been shown to possess unique anti-cancer activity. In this study, we have deeply investigated the therapeutic effect of elaiophylin on LADC and found elaiophylin exerts its anti-cancer effect though inhibiting mitophagy and oxidative stress and targeting SIRT1/Nrf2 signaling. This innovative and comprehensive research may provide the possibility for the development of novel chemotherapy drug for LADC.

**Abstract:**

Lung adenocarcinoma (LADC), the most common type of lung cancer, is still one of the most aggressive and rapidly fatal tumor types, even though achievements in new therapeutic approaches have been developed. Elaiophylin as a C2 symmetrically glycosylated 16 macrolides has been reported to be a late-stage autophagy inhibitor with a potent anti-tumor effect on various cancers. This study investigated the anti-tumor effect of elaiophylin on human LADC for the first time in in vitro and in vivo models. The in vitro study in LADC A549 cells showed that elaiophylin significantly inhibited cell viability and induced cell apoptosis through the suppression of mitophagy and induction of cellular and mitochondrial oxidative stress. Proteomic analysis and molecular docking assay implicated that SIRT1 was likely the direct target of elaiophylin in A549 cells. Further mechanistic study verified that elaiophylin reduced Nrf2 deacetylation, expression, and transcriptional activity as well as cytoplasm translocation by downregulating SIRT1 expression and deacetylase activity. Additionally, SIRT1/Nrf2 activation could attenuate elaiophylin-induced mitophagy inhibition and oxidative stress. The in vivo study in the A549-xenograft mice model showed that the anti-tumor effect of elaiophylin was accompanied by the decreased expressions of SIRT1, Nrf2, Parkin, and PINK1. Thus, the present study reports that elaiophylin has potent anti-tumor properties in LADC, which effect is likely mediated through suppressing the SIRT1/Nrf2 signaling. In conclusion, elaiophylin may be a novel drug candidate for LADC and SIRT1 may be a new therapeutic target for such devastating malignancy.

## 1. Introduction

Lung adenocarcinoma (LADC) is the most common type of lung cancer, accounting for about 40% of all lung cancers [1]. Despite new therapeutic approaches that have been developed for LADC, it remains one of the most aggressive and fatal tumor types. Chemotherapy still remains the primary therapeutic strategy for this malignancy. However, the arising drug resistance becomes the major challenge of its treatment effectiveness. Therefore, it is emerged to discover and develop new chemotherapeutic agents with improved efficacy and specificity.

Elaiophylin is a C2 symmetrically glycosylated 16-membered macrolide, originally isolated from *Streptomyces* melanophores [2]. Elaiophylin has been shown to possess unique and extensive pharmacological activities including anti-bacterial, anti-helminth, anti-cancer, anti-virus, and immunosuppressive effects [3,4]. In recent years, research endeavors have been made to explore its anti-cancer effect. Haet et al. have reported that elaiophylin can inhibit tumor cell-induced angiogenesis in in vitro and in vivo models by downregulating the expression of vascular endothelial growth factor (VEGF) and inhibiting hypoxia-inducible factor-1α (HIF-1α) accumulation in human umbilical vein endothelial cells [5]. It has also been shown that elaiophylin is a late inhibitor of autophagy, which can block the autophagy flux and consequently result in the accumulation of autophagosomes in the multiple myeloma with mutant *TP53* and ovarian cancer [6,7]. In addition to protein aggregates, mitochondria can be targeted for selective autophagic degradation by virtue of ubiquitin tags on their outer surfaces, and this autophagic process is commonly referred to as the PINK1–Parkin pathway of mitophagy. Our previous study has shown that elaiophylin exhibits outstanding anti-cancer activity in human uveal melanoma (UM) cell lines and human UM primary cells through suppressing mitophagy, inducing oxidative stress leading to autophagic cell death [8]. Elaiophylin acts on SIRT1 in uveal melanoma cells and consequently impacts on the deacetylation and mitochondrial localization of FoxO3a [8]. Accumulating evidence has indicated that SIRT1 is a key regulator of oxidative stress and autophagic lysosomes in maintaining the stability of mitochondria; however, the role of SIRT1 in LADC remains unclear [9,10]. Therefore, this study aimed to explore the anti-cancer property of elaiophylin in LADC using in vitro and in vivo models as well as the involvement of SIRT1-related signaling in such an effect of elaiophylin.

## 2. Materials and Methods

### 2.1. Cell Lines and Reagents

The human lung adenocarcinoma cell lines A549, H1975, Calu-3, and human fibroblast cell line MRC-5 were obtained from the National Collection of Authenticated Cell Cultures (Shanghai, China). Cells were cultured in Dulbecco’s Modified Eagle’s Medium (DMEM, high glucose) with 10% (*v*/*v*) fetal bovine serum (FBS) in a humidified atmosphere of 5% CO_2_ at 37 °C. Elaiophylin was kindly provided by Prof. Yunying Xie (Institute of Medicinal Biotechnology, Chinese Academy of Medical Sciences and Peking Union Medical College, Beijing, China) with a purity of >99%. Elaiophylin was dissolved in dimethyl sulfoxide (DMSO) and stored at −20 °C before use.

### 2.2. Cell Viability Assay

MTT(3-(4,5-dimethylthiazol-2-yl)-2,5-diphenyltetrazolium bromide) assay was used to determine cell viability [11]. Firstly, Cells were seeded in 96-well plates at density of 1 × 10^4^ cells/well overnight. Culture medium with 5‰ DMSO was used as control group (elaiophylin, 0 µM). For MTT assay, cells were incubated with 5 mg/mL MTT solution for 4 h at 37 °C. Following that, 150 µL of DMSO was added to each well and the plates were mixed on an orbital shaker for 10 min at room temperature. The absorbance was measured at 490 nm using a SpectraMaxM5 microplate reader (Molecular Devices, San Jose, CA, USA).

### 2.3. Colony Formation Assay

A colony formation assay was adopted to assess reproductive cell death. Cells were seeded into 6-well plates and cultured for 14 days in a humidified atmosphere of 5% CO_2_ at 37 °C. Elaiophylin treatment was applied every 3 days. Cells were then fixed in 4% paraformaldehyde (Beyotime, Nantong, China) for 15 min at room temperature. After washing with PBS three times, cell colonies were stained with crystal violet for 15 min at room temperature followed by washing with PBS three times. Upon air drying the plates, the number of colonies was counted using an inverted phase contrast microscope (Olympus IX53, Tokyo, Japan).

### 2.4. Cell Apoptosis Assay

Cell apoptosis was measured with AnnexinV-FITC/PI double staining detection kit (Absin, Shanghai, China). After indicated treatment, cells were incubated with 300 μL binding buffer (containing 10 μL Annexin V-FITC and 10 μL PI) for 15 min in the dark at room temperature. The samples were analyzed with flow cytometry (Becton-Dickinson, San Jose, CA, USA) immediately and data were analyzed using FlowJo software (Becton-Dickinson, CA, USA).

### 2.5. Detection of Intracellular ROS

Intracellular ROS production was detected by DCFH-DA kit (Beyotime, Nantong, China). DCFH-DA is a cellular infiltrating and non-fluorescent dye that can react with the carboxy dichlorofluorescein produced by ROS and generate fluoresce signals. After indicated treatment, cells were incubated with DCFH-DA (10 μM) for 30 min in the dark at 37 °C. After washing twice with D-PBS, the signal was detected using a fluorescence microscope (Olympus IX53, Olympus Corporation, Tokyo, Japan).

### 2.6. Detection of Mitochondrial ROS

Mitochondrial ROS production was detected by Mitosox mitochondrial superoxide indicator (Warbio, Nanjing, China). After indicated treatment, cells were incubated with Mitosox (10 μM) for 30 min in the dark at 37 °C. After washing twice with D-PBS, the signaling was detected with a fluorescence microscope.

### 2.7. Mitochondrial Isolation

Mitochondria was isolated from A549 cells with the Cell Mitochondria Isolation Kit (Beyotime, Nantong, China) according to the manufacturer’s instructions. In brief, cells were collected and resuspended in ice-cold PBS and centrifuged (600 g, 5 min, 4 °C). Then, cells were resuspended in mitochondrial isolation buffer on ice for 15 min. Cells were then homogenized and the homogenate was centrifuged (600× *g*, 10 min, 4 °C). The supernatant was transferred to another tube and centrifuged (11,000× *g*, 10 min, 4 °C). The supernatant was carefully removed, and the pellet was resuspended with 150 μL of mitochondria storage buffer. The samples were either used immediately or stored at −80 °C [12].

### 2.8. Mitochondrial Membrane Potential Assessment

Mitochondrial membrane potential (MMP) was measured by Rhodamine 123 (Rh123) staining (Beyotime, Nantong, China). Rh123 can selectively enter the mitochondria at full membrane potential and remain in the mitochondria [13,14]. After indicated treatment, cells were incubated with 5 µg/mL Rh123 at 37 °C for 30 min in the dark. The samples were immediately analyzed at excitation wavelength of 507 nm and emission wavelength of 529 nm using a fluorescence microscope.

### 2.9. Mito-Keima Mitophagy Analysis

Cells were transfected with the mKeima-Red-Mito-7 plasmid using Lipofectamine 3000. After 24 h, cells were treated with different concentrations of elaiophylin for another 24 h. The cells were then imaged using a fluorescence microscope.

### 2.10. Immunofluorescent Assay

After indicated treatment, cells were cultured on glass coverslips for 24 h, fixed with 4% paraformaldehyde (PFA), and blocked with 5% bovine serum albumin (BSA) in PBS containing 0.4% Triton X-100. Subsequently, the cells were co-incubated with MitoTracker Green and primary antibodies at 4 °C overnight. Upon washing with PBS three times, cells were incubated with the fluorescent secondary antibody for 1 h at room temperature. After staining with DAPI for 5 min at room temperature, samples were washed with PBS for three times and mounted for visualization. The fluorescence signal was observed with a fluorescence microscope.

### 2.11. Proteomics and Bioinformatics Analysis

Cells treated with or without elaiophylin (0.25 μM, 24 h) were lysed in RIPA lysis buffer supplied with proteinase inhibitor cocktail. Protein concentration was measured using the BCA protein assay kit (Beyotime, Nantong, China) and protein samples were separated via sodium-dodecyl sulfate poly-acrylamide (SDS)-PAGE gel. Gels were stained with Coomassie brilliant blue staining (10% (*w*/*v*) ammonium sulfate, 1% (*v*/*v*) phosphoric acid, 0.1% (*w*/*v*) Coomassie blue) overnight. After that, gels were socked in the decolorizing solution (milli-Q water) for 6–8 h until a clear background was observed. The peptides were first diluted with 5 µL of diluted trypsin (diluted 0.1 mg/mL stock trypsin 1:10 into 25 mM ammonium bicarbonate) and then extracted twice by 15–25 µL of 50% acetonitrile, 5% trifluoroacetic acid (TFA) to each tube containing gel slice for 15 min as described in the literature [15]. Subsequently, the digested peptides were detected by mass spectrometry. The liquid phase was analyzed by EASY-nLC 1000 nano-upgraded ultra-high performance liquid chromatograph (Thermo Fisher, Waltham, MA, USA). The mobile phase A contains 0.1% formic acid aqueous solution, and the mobile phase B is MS grade acetonitrile. The SEQUEST algorithm of Proteome Discoverer 1.4 software (Thermo Fisher, MA, USA) was used for database retrieval. The DAVID, KOBAS, STRING databases, and Cytoscape software were adopted for GO function, KEGG pathway, and protein–protein interaction (PPI) analysis. The raw data have been uploaded to Sequence Read Archive (SRA) database (PRJNA898634).

### 2.12. Computational Docking Study

The binding effect of elaiophylin on SIRT1 allosteric site was predicted by molecular docking study. The ligand molecular structure was optimized using Gaussian 16 at the B3LYP/6-31G level. The crystal structure of the SIRT1 catalytic domain bound to nicotinamide adenine dinucleotide (NAD^+^) and an indole (EX527 analog) (4I5I) was acquired from protein database and then the molecules of water, NAD+, and EX527 analog in the crystal structure were removed using PyMOL software (https://pymol.org/2/ (accessed on 14 December 2021)). AutoDock Tools V.1.5.6 (http://autodock.scripps.edu (accessed on 16 December 2021)) was used to prepare the ligand and receptor as a PDBQT file [16,17]. The scoring matrix of this program adopts the default setting of AutoDock Vina 1.1.2 as random. Grid boxes with sizes of 28x, 36y, and 30z were set around the residues formed at the allosteric site of SIRT1 with centers at −43.326x, −19.256, and 20.249z, respectively. In the study, the ligand remained flexible, allowing all bonds to rotate freely, while the receptor was considered rigid. The results were analyzed according to docking score/binding energy and visualized using PyMOL software.

### 2.13. Western Blot Analysis

Cells after treatment were collected and dissolved in RIPA buffer (Beyotime, Nantong, China) and protein concentration was measured using the BCA protein assay kit. A total of 50 μg protein in each sample was isolated by 15% SDS-PAGE and then imprinted on PVDF membrane (Beyotime, Nantong, China). The membrane was blocked and incubated with primary antibodies overnight at 4 °C. The blots were incubated with HRP-conjugated secondary antibody at room temperature for 1 h on the next day. The ECL assay kit (Beyotime, Nantong, China) was used to reveal the protein bands. The density of each band was normalized to the expression of GADPH (Cat. No: ab8245, Abcam, Cambridge, MA, USA) or VDAC1 (Cat. No. ab15895, Abcam). The other primary antibodies used include Cytochrome c (Cat. No: ab13575, Abcam), SIRT1 (Cat. No. ab110304, Abcam), SIRT3 (Cat. No. ab217319, Abcam), LC3B (Cat. No. ab192890, Abcam), PINK1 (Cat. No. ab23707, Abcam), Parkin (Cat. No: ab77924, Abcam), Nrf2 (Cat. No. ab137550, Abcam), Nrf2 (Acetyl-Lys599) (Cat. No. HW147, Signalway Antibody, Nanjing, China). All experiments were repeated three times, and the gray values were quantified using Image J software (version 1.8.0 for Windows, National Institutes of Health, NY, USA).

### 2.14. Cell Transfection

The SIRT1 expression vector pcDNA3.1-SIRT1 and Nrf2 expression vector pcDNA3.1-Nrf2 were purchased from GenePharma (Shanghai, China). The SIRT1 siRNA was purchased from Santa Cruz Biotechnology (sc-40986, Dallas, TX, USA). Cells were seeded in 96-well plates (1 × 10^4^ cells/well) or 6-well plates (1 × 10^5^ cells/well) and transfected with empty or expression vector (1 μg/mL) or siRNA (50 nM) using Lipofectamine 2000 reagent (Invitrogen, Carlsbad, CA, USA) at 37 °C according to the manufacturer’s instruction. Forty-eight hours later, Western blot analysis was performed to examine the efficiency of transfection. Then, after transfection for 48 h, cells could be used for the subsequent experiments.

### 2.15. Dual-Luciferase Reporter Assay

Dual-luciferase report assay was conducted to assess the transcriptional activity of Nrf2. The human PINK1 ARE (5′-TGCTTGAGC-3′) and HMOX1 ARE (5′-CGGACCTTGACTCAGCAGAAAA-3′) were, respectively, inserted into the pGL3 vector (Promega, Madison, WI, USA) by Genepharma (Shanghai, China). The plasmid pRL-TK encoding Renilla luciferase was used as an internal control. Then, cells were co-transfected with pGL3 vector, pcDNA3.1-Nrf2, or internal control plasmid (pcDNA3.1-vector) by Lipofectamine 2000 reagent (Invitrogen, CA, USA) at 37 °C according to the manufacturer’s instruction in A549 cells. Additionally, the luciferase assay was performed 48 h after transfection using the Firefly/Renilla Dual-Luciferase Reporter Assay System (Promega, WI, USA) [18,19,20].

### 2.16. SIRT1 Enzyme Activity Assay

SIRT1 enzymatic activity was assessed by using commercial kits (ab156065) from Abcam plc. (Cambridge, UK) in accordance with the manufacturer’s instructions. First, assay buffer (50 mM TRIS-HCl, pH 8.0, 137 mM sodium chloride, 2.7 mM potassium chloride, 1 mM magnesium chloride, 1 mg/mL bovine serum albumin), SIRT1 enzyme, and either solvent (DMF) or different concentrations of elaiophylin were mixed with the co-substrate (NAD) for 45 min. Thereafter, the stop/developing solutions, containing a mixture of a developer, were added to the microplate and incubated for 30 min at 25 °C. The deacetylated peptide reacts with the developer and releases a fluorophore. The fluorophores in both assays were analyzed at an excitation wavelength of 350 nm and an emission wavelength of 450 nm. The inhibitory percentage of the samples on the SIRT1 enzyme activity was calculated as the ratio of fluorescent intensity between samples and vehicle control [21].

### 2.17. Nude Mice Tumorigenesis Assay

Animal studies were approved by the Laboratory Animal Ethics Committee of Jiangsu Institute of Nuclear Medicine (Wuxi, China). Luc-A549 cells (5 × 10^6^) were dissolved in the normal saline and then mixed with Matrigel at a 2:1 volume ratio. The mixture was injected subcutaneously (the right side of the hips) into 5-week-old BALB/c nude mice (Cavens Laboratory Animal Co., Ltd., Changzhou, China). When the tumor volumes reached approximately 100 mm^3^, the mice were divided randomly into two groups (*n* = 5 per group). The mice were then administered with vehicle or elaiophylin (2 mg/kg) by intraperitoneal injection once daily for 14 days. Body weight and tumor volumes were measured every other day. At the end of the treatment, each mouse was injected with 15 mg/mL luciferase potassium and then 4% chloral hydrate intraperitoneally. Fluorescence imaging was conducted using noninvasive bioluminescence in vivo imaging system (IVIS Spectrum, PerkinElmer, Shelton, CT, USA). After imaging, the tumors were removed, weighed, and photographed.

### 2.18. Histology and Immunohistochemistry

Tumor tissues were surgically isolated from each mouse and fixed in formalin, embedded in paraffin, and then sectioned. The sections were processed for H&E, Ki67, or TUNEL staining. For immunohistochemical staining, the sections were incubated with SIRT1 antibody overnight at 4 °C and then with HRP-conjugated secondary antibody for 2 h at room temperature. The sections were visualized using a DAB kit (Beyotime, Nantong, China), and the images were observed using a light microscope (Olympus IX53, Tokyo, Japan).

### 2.19. Statistical Analysis

IBM SPSS (version 19.0 for Windows, NY, USA) package was used to analyze the data. All data were presented as the mean ± SD for a minimum of three independent experiments and triplicates in each experiment. Statistical comparisons were conducted with the Student’s *t*-test between two groups and a one-way ANOVA followed by Tukey’s post hoc test among three groups. A value of *p* < 0.05 was considered as statistically significant.

## 3. Results

### 3.1. Elaiophylin Inhibits Cell Viability and Induces Cell Apoptosis in A549 Cells

**Figure 1:**
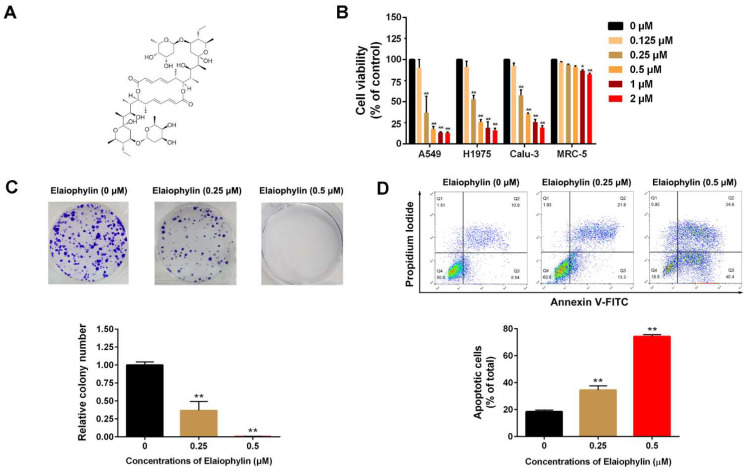
Elaiophylin inhibits cell viability and induces cell apoptosis in A549 cells. (**A**) The chemical structure of elaiophylin. (**B**) A549, H1975 ,Calu-3 and MRC-5 cells were treated with elaiophylin (ranged from 0 to 2 µM) for 24 h, and cell viability was determined by MTT assay. (**C**) A549 monoclonal cells were treated with elaiophylin for 24 h and then continuously cultured for 14 days. The formation of colonies was determined by crystal violet staining. (**D**) A549 cells were treated with elaiophylin (0, 0.25 and 0.5 µM) for 24 h, and cell apoptosis was determined by Annexin V/PI staining using flow cytometry. Apoptotic cell proportions were showed in bars. Data was expressed as means ± SD of three independent experiments and each experiment included triplicated repeats. * *p* < 0.05, ** *p* < 0.01 vs. control.

The chemical structure of elaiophylin is shown in Figure 1A. MTT and colony formation assays were used to investigate the cytotoxic effect of elaiophylin in human lung adenocarcinoma cells (A549, H1975, Calu-3). As shown in Figure 1B, elaiophylin treatment for 24 h dose-dependently inhibited cell viability of all three lung adenocarcinoma cell lines, but it had minimal cytotoxic effect on human fibroblast cell MRC-5. Among the three cell lines, A549 cells as the most commonly used cell line in LADC study, showed the most pronounced respond to elaiophylin, so which were selected for the subsequent experiments. It has been estimated that the IC_50_ of elaiophylin was 248.8 nM in A549 cells (data now shown). In addition, consistent results were obtained using colony formation assay in A549 cells (Figure 1C) and then it was selected for the subsequent mechanistic study. Further cell apoptotic analysis was performed using flow cytometry, in which results indicated that elaiophylin treatment for 24 h significantly induced an increased apoptosis of A549 cells (Figure 1D).

### 3.2. Elaiophylin Induces Intracellular and Mitochondrial ROS Production in A549 Cells

**Figure 2:**
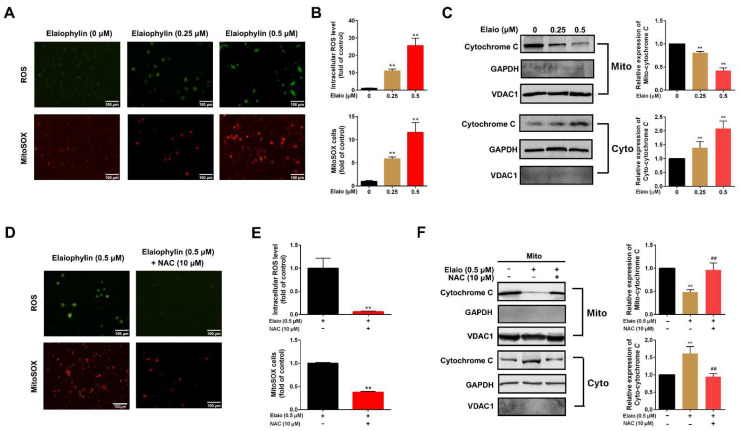
Elaiophylin induces intracellular and mitochondrial ROS in A549 cells. A549 cells were exposed to elaiophylin for 3 h with or without pre-treatment with NAC (10 μM) for 6 h. (**A**,**B**,**D**,**E**) Intracellular ROS level detection was assessed using DCFH-DA fluorescence probe; while mitochondrial ROS level detection was assessed using Mitosox red mitochondrial superoxide probe. (**C**,**F**) Cytochrome C translocation was determined by Western blot analysis. GAPDH and VADC1 were used as reference proteins in cytoplasm and mitochondria, respectively. Data were expressed as means ± SD of three experiments and each experiment included triplicated repeats. ** *p* < 0.01 vs. control, ^##^ *p* < 0.01 vs. Elaiophylin group. Original Western Blots can be found at Supplementary Materials.

It is well known that mitochondrial damage induces the release of reactive oxygen species, which promotes cell death. To assess the oxidative stress induced by elaiophylin, intracellular and mitochondrial ROS production were measured. As shown in Figure 2A,B, elaiophylin treatment for 3 h dose-dependently induced upregulation of both intracellular and mitochondrial ROS levels. According to the literature [22,23], the increase in superoxide-rich mitochondria may be the main cause of the production of damaged mitochondria. As shown in Figure 2C, mitochondrial damage was associated with the transfer of Cytochrome C from the mitochondria to the cytoplasm. In addition, the pre-treatment of an antioxidant agent NAC (N-acetyl-L-cysteine, 10 μM) significantly attenuated oxidative stress in A549 cells upon elaiophylin treatment (Figure 2D–F).

### 3.3. Elaiophylin Inhibits Mitophagy and Induces Mitochondrial Dysfunction in A549 Cells

**Figure 3:**
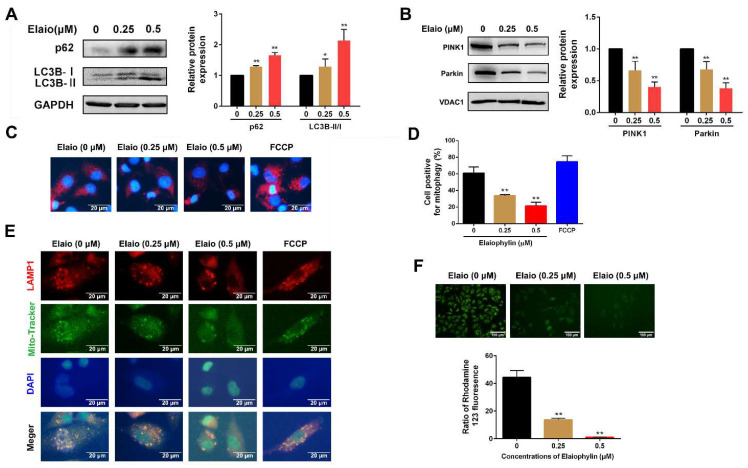
Elaiophylin inhibits mitophagy and induces mitochondrial dysfunction in A549 cells. A549 cells were exposed to elaiophylin or FCCP (a mitophagy agonist) for 24 h. (**A**) The expression of autophagy-related proteins (LC3B and SQSTM1 (p62)) was assessed by Western blot analysis. (**B**) The expressions of mitophagy-related proteins (PINK1 and Parkin) were assessed by Western blot analysis. (**C**,**D**) A549 cells overexpressing Mito-Keima plasmid were treated with elaiophylin for 24 h. Mito-Keima (red fluorescence) was detected by a fluorescence microscope. FCCP, a mitophagy agonist, was used as a positive control. (**E**) Co-localization of mitochondria and lysosomes was assessed by MitoTracker (200 nM) and LAMP1 co-staining. (**F**) Mitochondrial membrane potential was assessed by rhodamine 123 staining. Data were expressed as means ± SD of three experiments and each experiment included triplicated repeats. * *p* < 0.05, ** *p* < 0.01 vs. control. Original Western Blots can be found at supplementary materials.

To investigate the effect of elaiophylin on autophagy, the expressions of autophagy-related proteins LC3B and SQSTM1 (p62) were assessed. As shown in Figure 3A, elaiophylin treatment for 24 h significantly increased the change in LC3B and SQSTM1 (p62), which was consistent with the previous reports [6,7]. Mitophagy is a special type of autophagy that acts as a protective mechanism to remove excessive ROS generated by mitochondria. As shown in Figure 3B, 0.5 μM elaiophylin treatment for 24 h significantly decreased the expressions of PINK1 and Parkin (mitophagy markers). To further explore whether cytotoxicity induced by elaiophylin was associated with mitophagy inhibition, the Mito-Keima assay was used to analyze A549 cells transfected with mKeima-Red-Mito-7 plasmid. FCCP (an activator of mitophagy) was used as a positive control in this assay. As shown in Figure 3C,D, elaiophylin treatment for 24 h significantly reduced the fluorescence intensity in A549 cells, which suggested mitophagy suppression. To assess whether elaiophylin affected the fusion of autophagosomes in the mitochondria with lysosomes, the co-localization of mitochondrial (MitoTracker Green) and lysosomal (LAMP1) markers were analyzed. As shown in Figure 3E, elaiophylin treatment for 24 h significantly inhibited mitophagy in A549 cells with a reduced co-localization of mitochondria and lysosomes. Elaiophylin treatment for 24 h also significantly reduced the mitochondrial membrane potential in A549 cells as to rhodamine 123 staining (green fluorescence) assay (Figure 3F).

### 3.4. Proteomic Analysis and Molecular Docking for Target Prediction of Elaiophylin in A549 Cells

**Figure 4:**
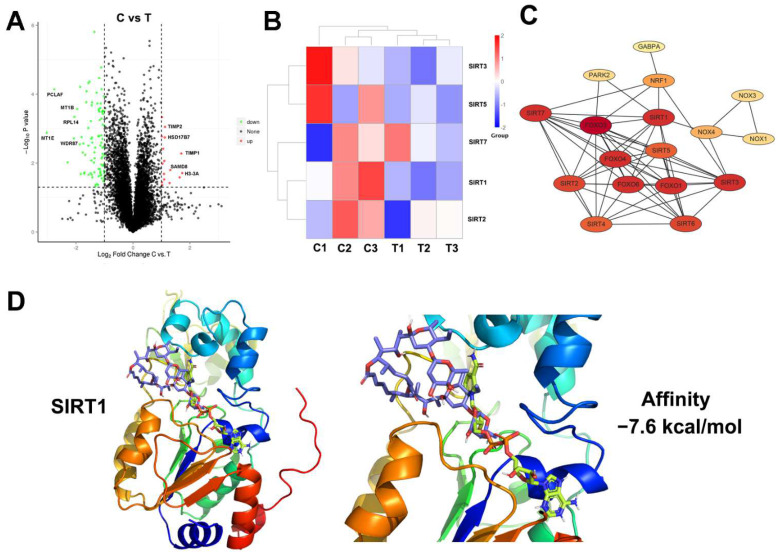
Proteomic analysis and molecular docking for target prediction of elaiophylin in A549 cells. (**A**) Volcano plots of differentially expressed proteins. (**B**) Heatmap analysis of SIRTs between elaiophylin treated group (0.25 μM) and control group. C: controls, T: treatment. (**C**) PPI network for the SIRTs. (**D**) Computational docking of elaiophylin and SIRT1 protein. NAD^+^: yellow sticks; elaiophylin: purple sticks.

Proteomic analysis and molecular docking were used for target prediction of elaiophylin in A549 cells. As shown in Figure 4A, there were 109 differentially expressed proteins between groups treated with or without elaiophylin, among which 17 proteins were upregulated, and 92 proteins were downregulated. However, as in KEGG and GO analysis, the autophagic pathway was slightly affected by elaiophylin treatment (Figure S1). Our previous study found that sirtuins-related proteins (SIRTs) are the molecular targets of elaiophylin in human uveal melanoma cells [8]. As to the result of proteomic analysis, SIRT4 and SIRT6 were not expressed, while SIRT1 expression was the highest and it was mostly affected by elaiophylin among all the seven SIRT isoforms (Figure 4B). In addition, PPI analysis showed that SIRT1 was a key molecule (Figure 4C). Molecular docking was further used to investigate whether elaiophylin is directly bound to the active site of SIRT1. As shown in Figure 4D, elaiophylin (purple sticks) was positioned out of the hydrophobic pocket, while competitively bound to the active binding site of nicotinamide adenine dinucleotide (NAD+, yellow sticks) in SIRT1 with the predicted binding affinity of −7.6 kcal/mol. Based on the above results, SIRT1 was likely to be the direct target of elaiophylin, which was further investigated in the subsequent mechanical experiments.

### 3.5. Elaiophylin Inhibits Mitophagy by Regulating SIRT1/Nrf2 Signaling in A549 Cells

**Figure 5:**
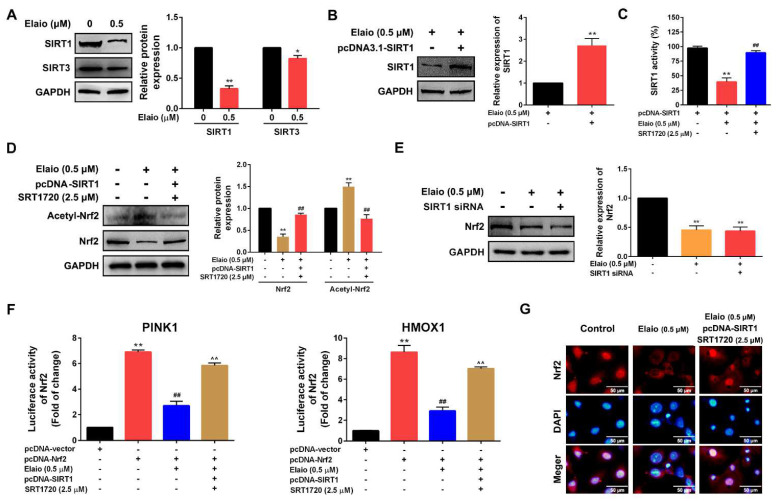
Elaiophylin inhibits mitophagy by regulating SIRT1/Nrf2 signaling in A549 cells. (**A**) A549 cells were exposed to elaiophylin (0.5 μM) for 24 h and the expression of SIRT1 and SIRT3 were assessed by Western blot analysis. (**B**) SIRT1 was overexpressed in A549 cells. (**C**) A549 cells overexpressing SIRT1 were pre-treated with or without SRT1720 (2.5 μM) for 24 h and exposed to elaiophylin (0.5 μM) for 24 h. The activity of SIRT1 was assessed by SIRT1 activity assay. (**D**) A549 cells with SIRT1 overexpression were pre-treated with or without SRT1720 (2.5 μM) for 24 h and exposed to elaiophylin (0.5 μM) for 24 h. The expressions of Nrf2 and Acetyl-Nrf2 were assessed by Western blot analysis. (**E**) A549 cells with or without SIRT1 knockdown were exposed to elaiophylin (0.5 μM) for 24 h. The expression of Nrf2 was assessed by Western blot analysis. * *p* < 0.05, ** *p* < 0.01 vs. control, ## *p* < 0.01 vs. elaiophylin group. (**F**) The transcriptional activity of Nrf2 was assessed by dual-luciferase reporter assay. ** *p* < 0.01 vs. control, ## *p* < 0.01 vs. A549 cells with Nrf2 overexpression group. ^^ *p* < 0.01 vs. elaiophylin+pcDNA-Nrf2 group. (**G**) Localization of Nrf2 was assessed by immunofluorescence staining. Data were expressed as means ±SD of three experiments and each experiment included triplicated repeats. Original Western Blots can be found at supplementary materials.

SIRT1 has been shown to be associated with oxidative stress [24,25]; thus, its involvement in the anti-mitophagy effect of elaiophylin was further investigated. As shown in Figure 5A, elaiophylin treatment for 24 h significantly downregulated the expressions of SIRT1 and SIRT3. The downregulation of SIRT1 was more obviously compared to SIRT3, and then SIRT1 was selected as the target for the subsequent experiments. Furthermore, cells overexpressing SIRT1 were treated with elaiophylin, and the deacetylase activity of SIRT1 was assessed. As shown in Figure 5B,C, SIRT1 activity was inhibited by elaiophylin treatment for 24 h, which in turn was reversed by a SIRT1 agonist (SRT1720). Nuclear factor-erythrocyte 2-associated factor 2 (Nrf2), an essential downstream target of SIRT1, is an important antioxidant sensor in the cellular defense mechanism [26,27]. As shown in Figure 5D–F, elaiophylin treatment for 24 h significantly inhibited Nrf2 deacetylation, downregulated Nrf2 expression in A549 cells, and reduced transcriptional activity of Nrf2 in Nrf2 overexpressed A549 cells; however, SIRT1 knockdown showed no effect on the expression of Nrf2 in cells with elaiophylin treatment. In addition, Nrf2 was more abundantly detected in the cytoplasm than in the nucleus upon elaiophylin treatment, indicating its deactivation. SIRT1 overexpression plus the pre-treatment of SRT1720 effectively attenuated the above effect (Figure 5G). All these observations suggested that elaiophylin impacts Nrf2 in a SIRT1-dependent manner.

### 3.6. Activation of SIRT1/Nrf2 Signaling Attenuates the Anti-Mitophagy Effect of Elaiophylin in A549 Cells

**Figure 6:**
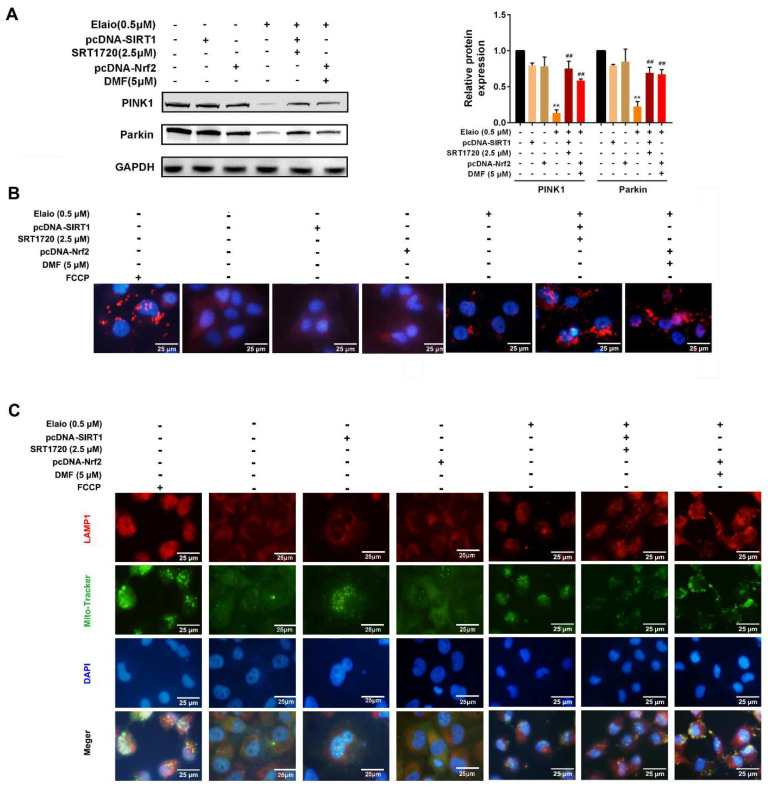
Activation of SIRT1/Nrf2 signaling attenuates the anti-mitophagy effect of elaiophylin in A549 cells. The cells were divided into six groups: control, SIRT1 overexpression only, Nrf2 overexpression only, elaiophylin, elaiophylin+SIRT1 activation (SIRT1 overexpression plus SRT1720), elaiophylin+Nrf2 activation (Nrf2 overexpression plus DMF). (**A**) The expressions of mitophagy-related proteins PINK1 and Parkin were assessed by Western blot analysis. (**B**) Cells overexpressing Mito-Keima were treated with elaiophylin for 24 h. Mito-Keima (red fluorescence) was assessed by a fluorescence microscope. FCCP, a mitophagy stimulant, was used as a positive control. (**C**) Colocalization of mitochondria and lysosomes was assessed by MitoTracker (200 nM) and LAMP1 staining. Data were expressed as mean ± SD of three experiments. ** *p* < 0.01 vs. control, ^##^ *p* < 0.01 vs. elaiophylin group. Original Western Blots can be found at supplementary materials.

**Figure 7:**
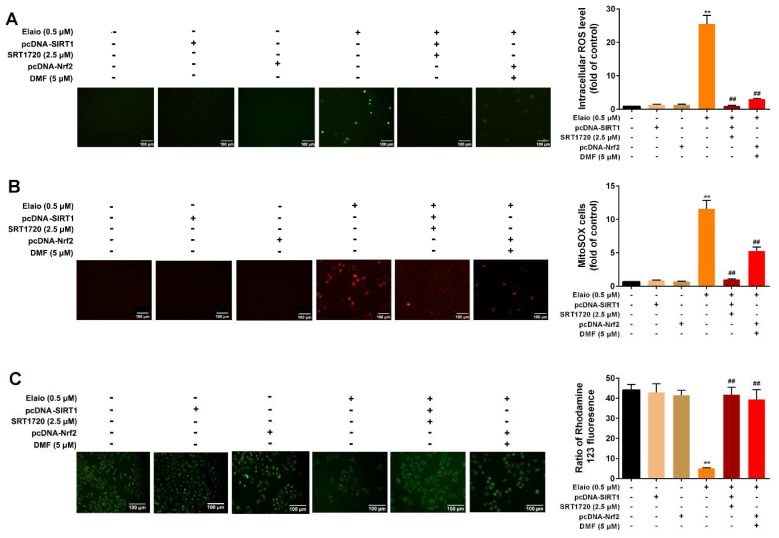
Activation of SIRT1/Nrf2 signaling attenuates the antioxidant effect of elaiophylin in A549 cells. The cells were divided into six groups: control, SIRT1 overexpression only, Nrf2 overexpression only, elaiophylin, elaiophylin+SIRT1 activation (SIRT1 overexpression plus SRT1720), elaiophylin+Nrf2 activation (Nrf2 overexpression plus DMF). (**A**,**B**) Intracellular ROS level was assessed using DCFH-DA fluorescence probe, and mitochondrial ROS level was evaluated using Mitosox Red fluorescence probe. (**C**) Mitochondrial membrane potential was assessed by rhodamine 123 staining. Data were expressed as mean ± SD of three experiments. ** *p* < 0.01 vs. control, ^##^ *p* < 0.01 vs. elaiophylin group.

The subsequent studies were performed to further confirm that elaiophylin directly targets SIRT1/Nrf2 signaling in A549 cells. The cells were divided into three groups: elaiophylin, elaiophylin+SIRT1 activation (SIRT1 overexpression plus SRT1720), elaiophylin+Nrf2 activation (Nrf2 overexpression plus DMF, an agonist of Nrf2). As shown in Figure 6A–C, activation of SIRT1/Nrf2 signaling significantly attenuated the anti-mitophagy effect of elaiophylin via increasing the expression of PINK1 and Parkin (mitophagy markers), enhancing the fluorescence intensity as shown in Mito-Keima mitophagy analysis and accelerating the fusion of autophagosome in the mitochondria with lysosomes. Additionally, Figure 7A–C showed that the activation of SIRT1/Nrf2 signaling also significantly reduced the intracellular and mitochondrial ROS and attenuated mitochondrial dysfunction upon elaiophylin treatment in A549 cells. In addition, SIRT1 overexpression only had no effect on mitophagy, ROS generation, and mitochondrial function.

### 3.7. Elaiophylin Suppresses Tumor Growth in a A549-Xenograft Model by Inhibiting SIRT1

**Figure 8:**
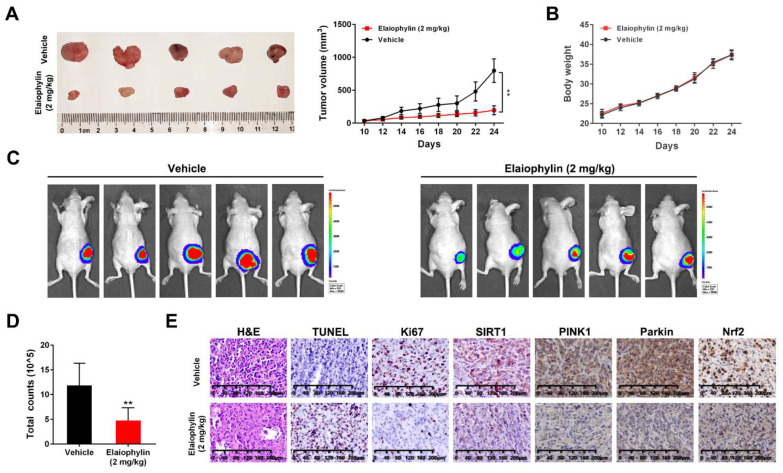
Elaiophylin suppresses tumor growth in an A549-xenograft model by inhibiting SIRT1. (**A**) After the elaiophylin treatment, the subcutaneous graft tumor was stripped and measured (*n* = 5). The image of tumor samples was shown on the left, and the volume change measured was shown on the right. (**B**) The weight changes in subcutaneously transplanted tumor mice with or without elaiophylin treatment were recorded. (**C**) In vivo fluorescence imaging of luc-A549 subcutaneous grafted tumors after fluorescein potassium injection. (**D**) The statistical analysis of fluorescence intensity in (**C**). (**E**) H&E staining of tumor sections of Luc-A549-xenograft mice treated with or without elaiophylin (the left panel). TUNEL assay (left middle panel) and immunohistochemistry staining of Ki67 (right middle panel) and SIRT1 staining (right panel) were conducted on tumor sections of Luc-A549-xenograft mice treated with or without elaiophylin. Data were expressed as means ± SD of three experiments and each experiment included triplicated repeats. ** *p* < 0.01 vs. control.

To investigate the in vivo effect of elaiophylin, the luc-A549 xenograft tumor model was intraperitoneally injected with elaiophylin (2 mg/kg) or vehicle for 14 consecutive days. Tumor volume and body weight were monitored every other day. The tumor volume in the elaiophylin treatment group was significantly smaller than that of the control group (Figure 8A); however, no significant changes in body weight were observed (Figure 8B). Fluorescein potassium salt injection was adopted for in vivo fluorescence imaging. As shown in Figure 8C,D, the fluorescence intensity of the elaiophylin treatment group was remarkably decreased. H&E staining showed that more inflammatory and necrotic cells were observed in the elaiophylin treatment group. Elaiophylin treatment significantly increased TUNEL-positive cells (brown color) and decreased proliferation-related indicator Ki67 (brown color). Importantly, elaiophylin significantly decreased the level of SIRT1 in in vivo models, which is consistent with our in vitro findings (Figure 8E).

## 4. Discussion

Mitophagy is a special type of autophagy, which plays a cellular protective role by clearing damaged mitochondria and reducing reactive oxygen species [28,29]. Mitophagy-mediated elimination of mitochondria is involved in many cellular processes including early embryonic development, cell differentiation, and apoptosis [30]. Accumulating evidence indicated that mitophagy functions and maintains health throughout life. Mitophagy defects are associated with various pathological processes such as neurodegeneration, heart failure, cancer, and aging [31]. Currently, there are limited reports about the relationship between mitophagy and LADC. Chang et al. have reported that high PINK1 expression (a marker of mitophagy) is an independent prognostic factor of LADC, which is correlated with poor response to chemotherapy [32]. In this study, we reported that mitophagy inhibition by elaiophylin could induce intracellular and mitochondrial ROS and suppress cell viability in in vitro and in vivo models of LADC. In addition, elaiophylin showed a slightly cytotoxic effect on human lung fibroblasts (MRC5), which were often used to evaluate the safety of anticarcinogen [33,34,35].

The Sirtuins family is a class of NAD^+^-dependent deacetylases that participates in a large number of important redox reactions, such as gluconeogenesis, glycolysis, tricarboxylic acid cycle, oxidative respiratory chain, etc. [36]. The dysregulation of the Sirtuins family plays an important role in various human prevalent diseases such as tumors, cardiovascular diseases, neuronal diseases, liver diseases, inflammation, and aging [32,37]. Sirtuins are known to control autophagy and mitophagy in cancers by acting on transcription factors or proteins related to autophagy and mitophagy mechanisms [38]. It is shown that increased ROS production occurs when cells are under stress. Additionally, the co-enzyme NAD activates sirtuins and regulates the activity of antioxidant reactive elements (AREs), which in turn regulates transcription of pro- and antioxidant genes to maintain redox cascades [39,40]. As for the previous literature, sirtuins have been linked to the control of autophagy and mitophagy by modulating transcription of autophagy and mitophagy genes, by post-translational modification of proteins belonging to the autophagy and mitophagy machinery [38]. Then, we put focus on the involvement of sirtuins in the elaiophylin’s anti-mitophagy effect and found that SIRT1 was the significantly regulated protein among sirtuins by proteomic assay and Western blot analysis. SIRT1, one of the most extensively and thoroughly studied sirtuins family proteins, is a key player in maintaining homeostasis against DNA damage, aging, and apoptosis under oxidative stress [39]. Then, molecular docking further clarified that SIRT1 might be the direct target of elaiophylin. Further analysis revealed that elaiophylin impacted the expression and deacetylase activity of SIRT1. Mitophagy relies heavily on two factors: the PTEN-induced putative kinase 1 (PINK1) and E3 ubiquitin ligase Parkin [41,42]. These proteins are responsible for sensing the function and health status of mitochondria and selecting damaged mitochondria for autophagy processing [43]. The link between SIRT1 and mitophagy was first demonstrated by Hwang’s group. They found that nicotinamide significantly extended the replication life of primary human fibroblasts by accelerating mitophagy degradation [44]. It was also shown that niacinamide-induced mitophagy is mediated by increased NAD^+^/NADH ratio and SIRT1 activation, and the SIRT1 activator SRT1720 can be used to mimic nicotinamide-induced mitochondrial phenotype [45]. Nowadays, more and more studies have evidenced that SIRT1 participates in the PINK1-Parkin labeled mitophagy process to regulate cellular oxidative stress, which may have therapeutic values [46,47,48,49]. To present, several studies reported that SIRT1 overexpression promotes the occurrence of LADC, which is closely related to its invasion and metastasis leading to poor prognosis [50,51,52]. Consistently, the current study indicated that elaiophylin increased the accumulation of autophagy surface marker protein LC3B and SQSTM1 (p62), decreased the expressions of PINK1 and Parkin, and inhibited the fusion of autophagosome in the mitochondria with lysosomes. However, SIRT1 overexpression only in A549 cells has no effect on the mitophagy. Meanwhile, SIRT1 overexpression plus SIRT1 activator SRT1720 treatment significantly attenuated the cellular effect of elaiophylin, indicating elaiophylin may exert its anti-mitophagy effect via regulating SIRT1 signaling. Future studies will investigate whether elaiophylin directly binds with SIRT1, probably using the SPR (surface plasmon resonance) method.

As a NAD-dependent deacetylase, SIRT1 regulates downstream cellular pathways in relation to oxidative stress by deacetylating various transcription factors to maintain cell survival [53,54]. Previous studies have observed that the SIRT1/Nrf2 signaling pathway is associated with various cellular responses related to oxidative stress [55,56]. Recently, it has been reported that Nrf2 is an important downstream target of SIRT1. Nrf2 is known to be an important antioxidant sensor in cellular defense mechanisms, responding to oxidative damage [57,58]. In normal cells, activation of NRF2 helps prevent the initiation of cancer by chemical carcinogens; however, in many tumor types, NRF2 is permanently upregulated and its overexpressed target genes support the promotion and progression of cancer by suppressing oxidative stress [59]. Activation of Nrf2 has also been shown to protect cells from oxidative damage induced by electrophilic compounds [60]. Once Nrf2 is activated by electrophilic compounds, it translocates to the nucleus and binds to the electrophilic response element (ERE), which further regulates the proteins involved in electrophilic detoxification and elimination, thereby enhancing the cell’s antioxidant capacity [61]. It was noticed that SIRT1 significantly enhanced Nrf2 pathway activity and inhibited ROS overproduction by decreasing the expression of heme oxygenase 1(HO-1) to facilitate Nrf2 translocating to the cytoplasm [56,60]. A large number of studies have found that Nrf2 is highly expressed in A549 cells. Targeting Nrf2 activity can inhibit the growth of LADC and eliminate the resistance to chemotherapy [62,63,64]. In this study, we found that elaiophylin inhibited the deacetylation of Nrf2, leading to reduced stability of Nrf2 by promoting Nrf2 entering the cytoplasm. In addition, the reduced expression and transcriptional activity of Nrf2 were in a SIRT1-dependent manner. However, Nrf2 overexpression only in A549 cells has no effect on mitophagy. In addition, Nrf2 overexpression plus Nrf2 activator DMF treatment could significantly attenuate the anti-mitophagy effect of elaiophylin. Compared to the previous study in UM, we found that SIRT1 was the direct target of elaiophylin by molecular docking and the SIRT1-Nrf2 pathway was activated in regulating mitophagy upon elaiophylin, which was not consistent with the previous study. However, there are still some limitations to this study. First, surface plasmon resonance or other methods need to be conducted to confirm the direct interaction of elaiophylin and SIRT1, and then an orthotopic transplanted mouse model needs to be constructed in the further study which is closer to the environment of the tumor.

**Figure 9:**
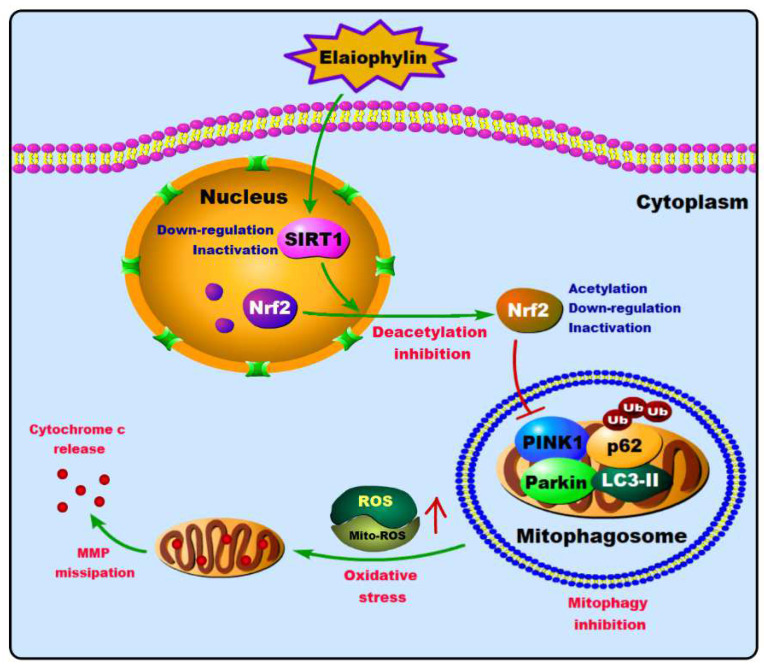
The proposed molecular mechanism of elaiophylin on human lung adenocarcinoma cells.

Overall, our data suggested that elaiophylin has a potent cytotoxic effect on LADC by inhibiting mitophagy and increasing oxidative stress. Specifically, elaiophylin exerted its effect by suppressing the deacetylation of Nrf2 in a SIRT1-dependent manner, leading to the increase in non-functional Nrf2 in the cytoplasm (Figure 9). Therefore, as a new autophagy inhibitor, elaiophylin may be developed into a new therapeutic regimen for LADC. Of course, whether it can be used for clinical treatment still needs further research.

## Data Availability

The original contributions presented in the study are included in the article/Supplementary Material, further inquiries can be directed to the corresponding authors.

## Supplementary Materials

The following supporting information can be downloaded at: https://www.mdpi.com/article/10.3390/cancers14235812/s1, Figure S1: (A) KEGG analysis between elaiophylin-treated group (0.25 μM) and control group. (B) GO enrichment analysis between elaiophylin treated group (0.25 μM) and control group; supplementary file: Original Western Blots and raw data.
