# Supplementary material for: Elaiophylin Inhibits Tumorigenesis of Human Lung Adenocarcinoma by Inhibiting Mitophagy via Suppression of SIRT1/Nrf2 Signaling
Source: Cancers (Basel). 2022 Nov 25;14(23):5812. doi: 10.3390/cancers14235812 (PMC9737501; doi:10.3390/cancers14235812)
Supplement: Supplementary file 1 [file cancerns-1966701 Figure S1.pdf › cancerns-1966701 Figure S1.pdf]

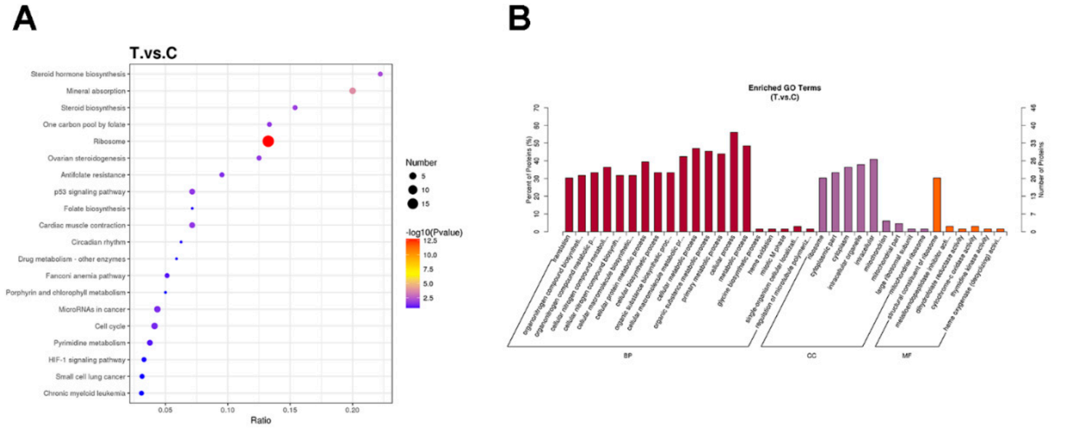

Figure S1. (A) KEGG analysis between elaiophylin-treated group (0.25 μM) and control group. **(B)** GO enrichment analysis between elaiophylin treated group (0.25 μM) and control group.
