# Supplementary material for: Elaiophylin Inhibits Tumorigenesis of Human Lung Adenocarcinoma by Inhibiting Mitophagy via Suppression of SIRT1/Nrf2 Signaling
Source: Cancers (Basel). 2022 Nov 25;14(23):5812. doi: 10.3390/cancers14235812 (PMC9737501; doi:10.3390/cancers14235812)

Figure 2C

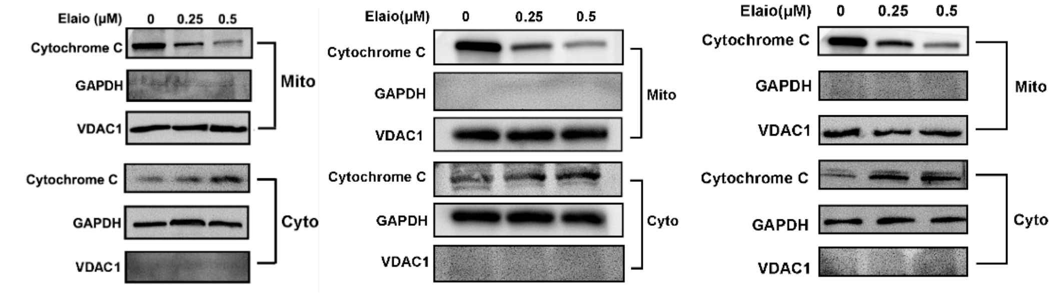

Figure 2F

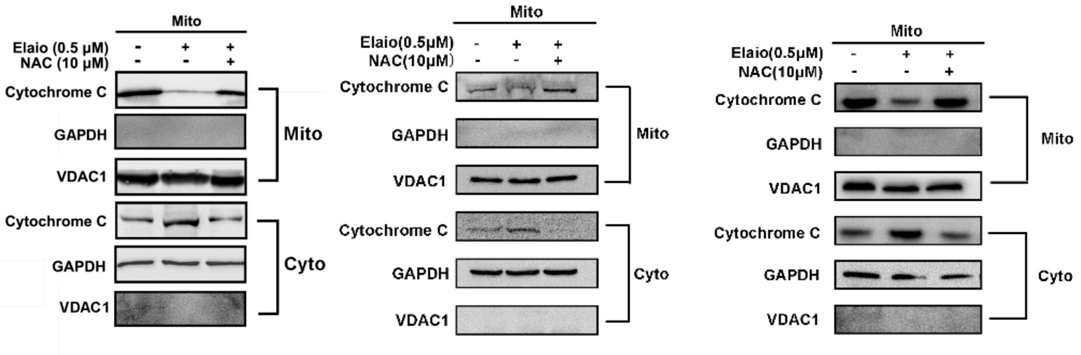

Figure3A

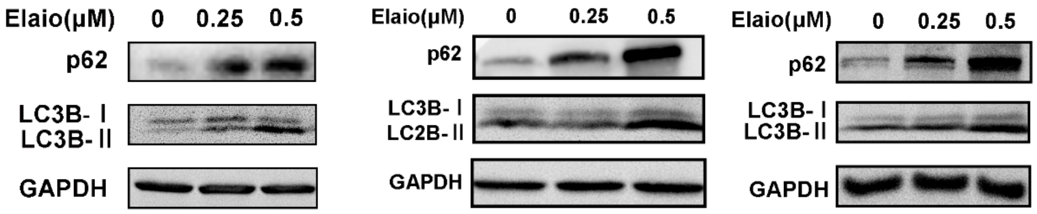

Figure3B

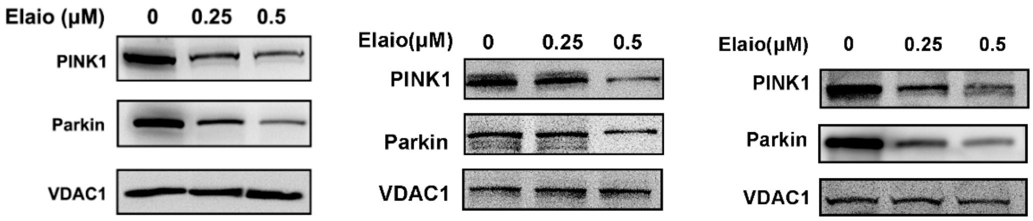

Figure5A

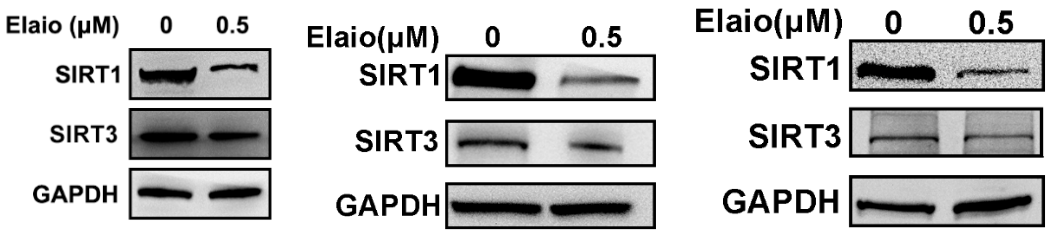

Figure 5B

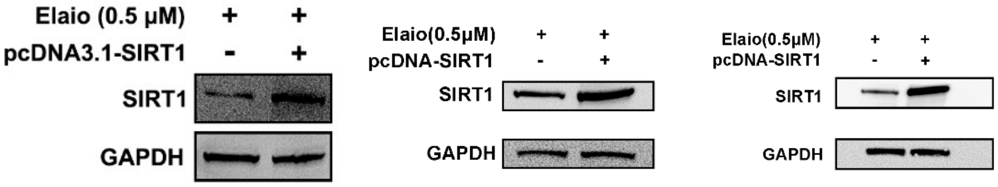

Figure 5D

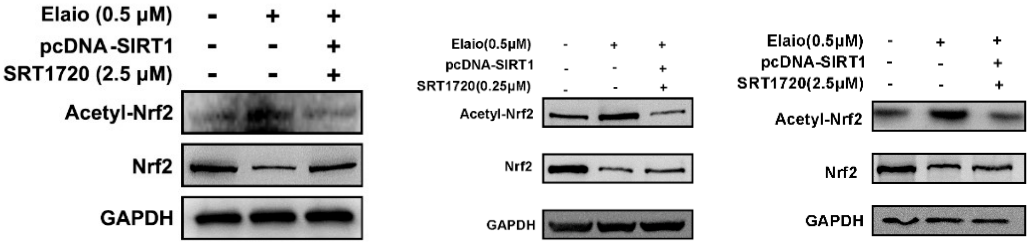

Figure 5E

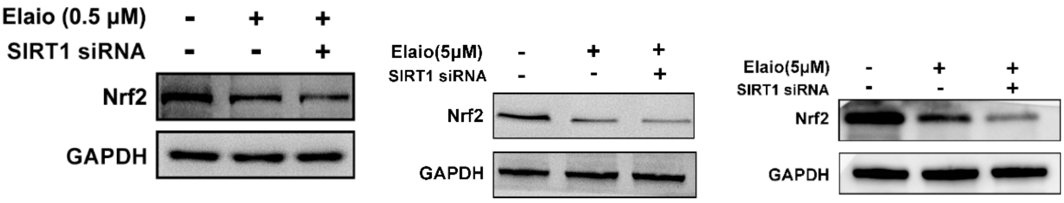

Figure 6A

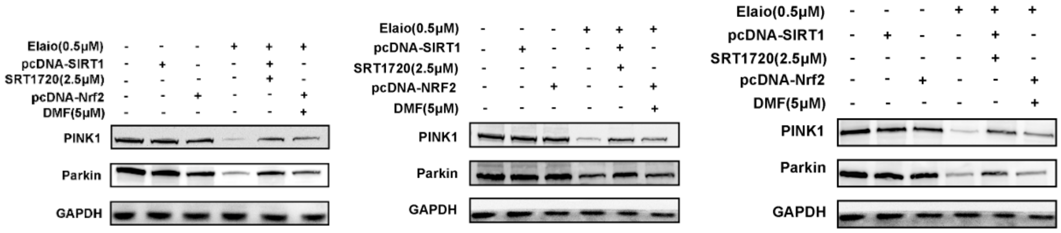

Supplement: Supplementary file 1 [file cancers-14-05812-s001.zip › cancers-1966701 supplementary file.zip!/Original Western Blots.pdf]
